# Multifunctional CMC–Nanographene Oxide Hydrogels Couple Fluorophore-Free Cancer Cell Imaging with Antioxidant and Antimicrobial Activity

**DOI:** 10.3390/pharmaceutics18091121

**Published:** 2026-09-07

**Authors:** Jordane S. Rodrigues, Sofia O. D. Duarte, Micheli de Souza Bernardes, Paola Pirela, Beatriz Ruivinho, Filipa Ramos, Rafael Parada Savino, Andressa Raianny Silva Soares, Pedro Henrique Gomes Araújo, Fernanda Guerra Lima Medeiros Borsagli, Pedro Fonte

**Affiliations:** 1 Institute of Engineering, Science and Technology, Universidade Federal dos Vales do Jequitinhonha e Mucuri/UFVJM, Av. 01, 4050 Cidade Universitária, Janaúba 39440-039, MG, Brazil; 2 iBB—Institute for Bioengineering and Biosciences, Department of Bioengineering, Instituto Superior Técnico, Universidade de Lisboa, Av. Rovisco Pais 1, 1049-001 Lisboa, Portugal; 3 Associate Laboratory i4HB—Institute for Health and Bioeconomy, Instituto Superior Técnico, Universidade de Lisboa, Av. Rovisco Pais, 1049-001 Lisboa, Portugal; 4 Department of Chemistry and Pharmacy, Faculty of Sciences and Technology, University of Algarve, Gambelas Campus, 8005-139 Faro, Portugal; 5 Centro de Ciências do Mar do Algarve (CCMAR/CIMAR LA), Campus de Gambelas, Universidade do Algarve, 8005-139 Faro, Portugal

**Keywords:** carboxymethyl cellulose, nanographene oxide, multifunctional hydrogels, intrinsic fluorescence, fluorescence-assisted bioimaging, drug-free biomaterials, antioxidant activity, antimicrobial activity

## Abstract

**Background:** The development of sustainable biomaterials that combine imaging capability with complementary biological functions without relying on incorporated drugs or exogenous fluorophores represents an attractive strategy for multifunctional biomedical platforms. **Methods:** Here, carboxymethyl cellulose (CMC) hydrogels reinforced with nanographene oxide (nGO) were developed by citric acid-mediated crosslinking using nGO contents of up to 3% (*w*/*w*) and evaluated for their physicochemical, electrochemical, and biological properties. **Results:** nGO exhibited a nanosheet morphology, a hydrodynamic diameter of 5.0 ± 0.2 nm, and a specific surface area of approximately 650 m^2^ g^−1^. Incorporation of nGO modified the hydrogel structure and reduced water uptake within the tested formulation window. The nanocomposite hydrogels maintained high cytocompatibility, with approximately 100% HeLa cell viability after 24 h. Confocal microscopy and quantitative fluorescence-intensity analysis demonstrated enhanced fluorescence relative to untreated cells, reaching approximately eightfold higher signal intensity and supporting proof-of-concept fluorescence-assisted visualization of HeLa cells without exogenous fluorescent probes. However, because only HeLa cells were evaluated and no cancer-specific targeting ligand was incorporated, these findings do not establish diagnostic specificity. The CMC–nGO hydrogels also exhibited radical-scavenging activity of up to 96.5% and antimicrobial activity against *Escherichia coli* and *Candida albicans*, with maximum inhibition zones of 12.9 and 9.2 mm, respectively. In addition, nGO incorporation modified the electrochemical response of graphite electrodes, supporting the multifunctional character of the platform. **Conclusions:** Overall, CMC–nGO hydrogels provide a sustainable, drug-free, and fluorophore-free biointerface combining fluorescence-assisted cell visualization with antioxidant, antimicrobial, and electrochemical functionalities. Further photophysical characterization, comparison with non-cancerous cervical cells, rheological and mechanical evaluation, and validation in advanced biological models are required to establish their potential for future bioimaging and biosensing applications.

## 1. Introduction

Cervical cancer remains a major global health burden and is predominantly associated with persistent infection with high-risk human papillomavirus (HPV) strains [1,2]. Despite established cytology- and HPV-based screening programmes, limitations related to accessibility, invasiveness, cost, and applicability to specific populations continue to motivate the development of complementary approaches for earlier and more accessible detection [2,3,4,5,6,7,8]. In parallel, increasing evidence links vaginal dysbiosis, oxidative stress, and local inflammation with cervical carcinogenesis. Microorganisms such as *Escherichia coli* and *Candida albicans* may contribute to disruption of the protective vaginal microbiota and to an altered local microenvironment [9,10,11]. These observations support interest in multifunctional biomaterials that combine imaging-related functions with biologically protective activities.

Hydrogel nanocomposites are attractive biomedical platforms because their hydrated polymer networks can accommodate functional nanomaterials while maintaining favourable cell–material interactions and molecular transport [12,13,14,15,16,17]. Among carbon-based nanomaterials, graphene oxide (GO) and nanographene oxide (nGO) offer a combination of high surface area, oxygen-containing functional groups, electrochemical responsiveness, intrinsic luminescence, and reported antioxidant and antimicrobial properties [18,19,20,21,22]. These characteristics have stimulated their investigation in biosensing and fluorescence-assisted bioimaging, including studies using HeLa cells as an in vitro cervical cancer model [23,24,25]. More broadly, advances in nanotechnology have enabled such multifunctional nanoscale materials to integrate optical, chemical, and biological functions within a single biomaterial platform [19].

Carboxymethyl cellulose (CMC) is a biocompatible and biodegradable cellulose derivative with hydroxyl and carboxyl groups that facilitate crosslinking and interactions with carbon-based nanomaterials [26,27,28,29,30,31,32,33]. Previous studies have explored CMC hydrogels containing carbon nanomaterials for drug delivery, biosensing, and bioimaging [34,35,36,37]. However, CMC–nGO hydrogels that simultaneously combine fluorescence-assisted cell visualization with antioxidant and antimicrobial functions remain comparatively underexplored, particularly in cervical cancer-related applications [38]. In this context, CMC can provide the hydrated structural matrix, whereas nGO may contribute complementary optical, radical-scavenging, antimicrobial, and electrochemical properties.

We therefore hypothesized that incorporation of nGO into citric acid-crosslinked CMC hydrogels would generate an intrinsically multifunctional biointerface capable of supporting fluorescence-assisted visualization of HeLa cells while providing antioxidant and antimicrobial activities. Because the photophysical origin and efficiency of the observed fluorescence were not fully characterized in the present study, the imaging component is considered a proof of concept rather than evidence of diagnostic specificity. Future photoluminescence characterization and comparison with non-cancerous cervical cells will be required to establish the origin, selectivity, and diagnostic relevance of the signal. Surface functionalization of nGO with disease-specific recognition moieties could subsequently provide a route towards selective fluorescence-assisted detection [7,39,40,41,42,43,44].

Accordingly, this study aimed to develop CMC–nGO hydrogels with different nGO loadings and evaluate their morphology, chemical structure, swelling behaviour, gel fraction, electrochemical response, cytocompatibility, fluorescence-assisted HeLa cell imaging, radical-scavenging activity, and antimicrobial activity against *E. coli* and *C. albicans*. By examining these complementary properties within a controlled formulation series, the work sought to establish composition–property relationships and assess the potential of CMC–nGO hydrogels as sustainable, drug-free and fluorophore-free biointerfaces for future bioimaging, biosensing, and cervical cancer-related biomedical applications.

## 2. Materials and Methods

### 2.1. Materials

Sodium salt of CMC (≥99.5%, DS = 0.7, Mw = 250,000 g.mol^−1^, Synth, Diadema, Brazil), citric acid (C_6_H_8_O_7_, ≥99.5%, Synth, Brazil), graphene oxide (≥99%, Carbon Explore, Campo Mourão, Brazil), 2,2-diphenyl-1-picrylhydrazyl (DPPH, Sigma-Aldrich, Darmstadt, Germany, 99%). Deionized water (DI-water) (Millipore Simplicity™, Merck, Darmstadt, Germany) with an 18 MW·cm resistivity was used.

### 2.2. Preparation of CMC–nGO Hydrogels

The hydrogel production process used sodium salt CMC and citric acid, with nGO incorporated at the concentrations shown in Table 1. Graphene oxide (Carbon Explore, ≥99% purity, Brazil) was produced by electrochemical exfoliation of graphite, with supplier-reported thickness < 2 nm, diameter 1.0–5.0 nm, and density 0.3 g cm^−3^. A 2% (*w*/*v*) CMC concentration was selected as a fixed formulation condition because it yielded a continuous, handleable network under the citric-acid crosslinking protocol while remaining sufficiently processable for nGO dispersion; this concentration is also consistent with the CMC hydrogel formulation framework used in our previous studies [4]. The aim of the present formulation series was therefore to isolate the effect of nGO loading rather than optimize CMC concentration. The 2% (*w*/*v*) CMC solution was stirred for 24 h, after which nGO was added and the dispersion was mixed at 4000 rpm for 24 h to maximize macroscopic homogeneity. This mixing time was selected from preliminary visual and SEM-based assessment of dispersion consistency. CMC molecular weight and viscosity were not measured before and after this high-speed mixing step; consequently, shear-induced chain scission cannot be excluded and should be directly evaluated in future process-optimization studies.

The nGO loading range was set to 0–3% *w*/*w* relative to the polymer content. This concentration range was selected based on preliminary trials and literature reports indicating that low-to-moderate nGO levels provide stable dispersions within hydrogels, prevent particle aggregation, and preserve cytocompatibility [4,41]. Concentrations above 3% were not tested because they are expected to compromise hydrogel homogeneity, increase cytotoxicity, and potentially quench fluorescence due to self-aggregation of graphene sheets. However, higher concentrations could, in principle, further enhance optical signals and antioxidant activity, albeit at the cost of biocompatibility.

**Figure 1:**
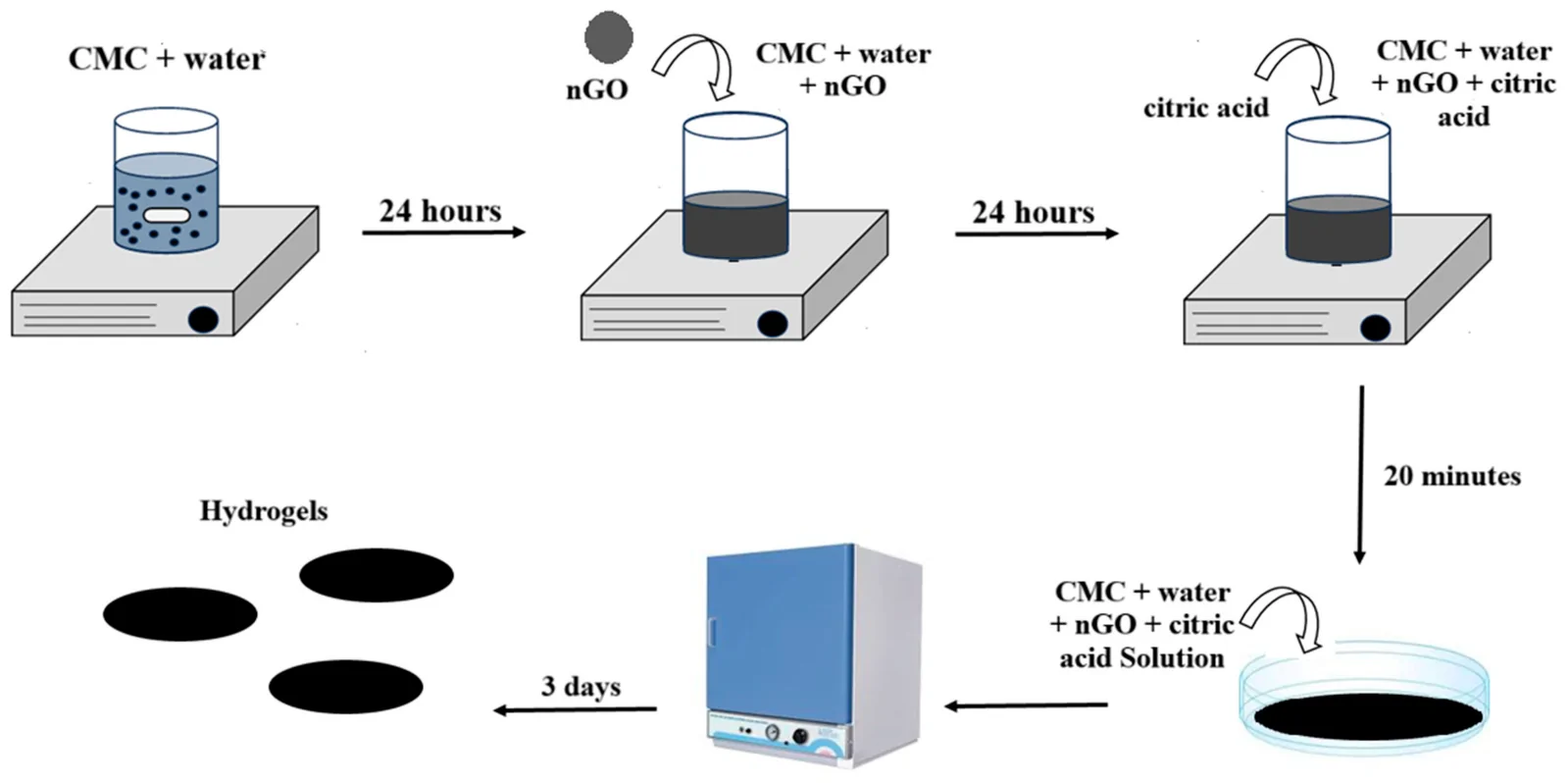
Schematic representation of the production of multifunctional CMC–nGO hydrogels. The process involves dispersion of nanographene oxide (nGO) within a carboxymethyl cellulose (CMC) solution, followed by citric acid-mediated crosslinking and thermal curing, resulting in a three-dimensional nanocomposite hydrogel network with embedded bioactive nanostructures.

Then, 15% (*w*/*w* relative to CMC) of citric acid was added to the solution and stirred for 20 min. Subsequently, the solution was transferred to a polystyrene Petri dish and placed in an oven set at 40 °C for 24 h to facilitate water removal. After, the oven temperature was increased to 80 °C for 24 h to crosslink the citric acid with the CMC chain, forming the hydrogel (Figure 1).

### 2.3. Characterization of CMC–nGO Hydrogels

The physicochemical and structural properties of the CMC–nGO hydrogels were evaluated using SEM, Fourier-transform infrared (FTIR) spectroscopy, X-ray diffraction (XRD), and swelling degree and gel fraction measurements, as described below.

#### 2.3.1. Scanning Electron Microscopy

SEM analysis was performed in a Zeiss Ultra Plus electron microscope (Carl Zeiss Microscopy GmbH, Jena, Germany) with accelerating 2–6 kV voltages at a working distance between 4 and 5 mm. The hydrogels underwent a sputtering process to coat them with a thin carbon film at a low deposition rate, ensuring no damage to the sample. The resulting film had a thickness of 30 nm. Secondary Electron (SE) images were acquired using an accelerating voltage of 15 kV.

#### 2.3.2. Fourier-Transform Infrared Spectroscopy

FTIR analysis was performed on a Nicolet 6700 device (Thermo Fisher, Waltham, MA, USA) using Attenuated Total Reflectance (ATR) in the wavelength range of 675 to 4000 cm^−1^, employing 32 scans and a resolution of 4 cm^−1^. Before the analysis, the samples were dried.

#### 2.3.3. X-Ray Diffraction

XRD analysis was conducted using an AXRD Benchtop Powder Diffractometer (PROTO, Taylor, MI, USA) with Cu-K*α* radiation (*λ* = 1.5406 Å). Measurements were performed in the 2*θ* range from 10.0075 to 80.0001° with steps of 0.002°.

#### 2.3.4. Swelling Degree and Gel-Fraction Analysis

To determine physiologic adsorption in tissue and the kinetic dissolution of the hydrogel, SD and GF procedures were performed as previously reported [35]. A PBS solution (pH = 7.0, room temperature) was used, and the hydrogels were analyzed at 1, 2, 3, 4, 6, 8, 24, and 48 h to reach equilibrium. In total, 28 samples were used for each system (*n* = 28; 7 samples of 4 different types: S0, S1, S2, and S3). Additionally, different pH values (5.0, 7.0, and 8.0) were analyzed to reflect different environments in the human body and changes that cancer cells can promote [45,46].

### 2.4. Analysis of nGO Properties

#### 2.4.1. Transmission Electron Microscopy and Energy-Dispersive X-Ray Spectroscopy

TEM coupled with EDX analyses were performed using an FEI Titan G2 80-300FEG S/TEM with a Schottky-type electron gun operated at 300 kV. EDX analysis was conducted using a Bruker XFlash 6T-30 detector with a resolution of 129 eV. The nGO was put in a copper grid. Secondary electron images were obtained at 300 kV for all samples (n ≥ 4).

#### 2.4.2. Raman Spectroscopy

Raman spectroscopy was conducted using a Cora 5001 equipment (Anton Paar, Graz, Austria) with a laser at an excitation wavelength (λ_exc_) of 532 nm and 50 W.

#### 2.4.3. Particle Size and Specific Surface Area

The particle size of nGO was determined by dynamic light scattering (DLS). For analysis, nGO was dispersed in deionized water at a concentration of 0.2% (*w*/*v*), and measurements were performed using a Zetasizer Nano ZS (Malvern Instruments Ltd., Malvern, UK). The selected dispersion concentration was based on preliminary measurements that provided an adequate scattering intensity and stable particle-size distribution. Complementary DLS measurements were performed using a NanoBrook 90Plus PALS instrument (Brookhaven Instruments Corporation, Holtsville, NY, USA) to confirm the particle-size distribution obtained with the primary instrument.

The specific surface area of nGO was determined by nitrogen adsorption using the multipoint Brunauer–Emmett–Teller method in a NOVA 1000 surface-area analyser (Quantachrome Instruments, Boynton Beach, FL, USA). Sample preparation, degassing conditions, measurement temperature, and the relative pressure range used for BET fitting should be reported to ensure reproducibility. Results were expressed as the mean ± SD of independent measurements.

### 2.5. Graphite Electrode Modification and Electrical Conductivity Assessment

The effect of CMC–nGO hydrogels on the electrochemical response of graphite electrodes was evaluated by cyclic voltammetry. A graphite working electrode with a geometric surface area of 0.068 cm^2^ was modified with a 1% (*w*/*v*) aqueous dispersion of each hydrogel formulation. The electrochemical behaviour of the modified electrodes was compared with that of the unmodified graphite electrode using a 1.0 mM hexaammineruthenium(III) solution, [Ru(NH_3_)_6_]^3+^, as the redox probe.

Electrochemical measurements were performed using a μAutolab III potentiostat and analysed with NOVA software, version 2.0. Cyclic voltammograms were recorded at a scan rate of 25 mV s^−1^ for 10 consecutive cycles in a conventional three-electrode electrochemical cell placed inside a Faraday cage and maintained at 25 °C. Before each measurement, the electrolyte solution was purged with nitrogen for 10 min to remove dissolved oxygen. Changes in peak current and voltammetric profile were used to assess the influence of hydrogel modification and nGO incorporation on electron-transfer behaviour and the electroactive surface of the graphite electrode.

### 2.6. Cytocompatibility Assessment of CMC–nGO Hydrogels

The cytocompatibility of the CMC–nGO hydrogels was evaluated in human cervical adenocarcinoma cells (HeLa; ATCC^®^ CCL-2™, Manassas, VA, USA) using the MTT assay. HeLa cells were cultured in Dulbecco’s modified Eagle medium (DMEM) supplemented with 10% (*v*/*v*) fetal bovine serum, 10 mg mL^−1^ streptomycin sulfate, 10 U mL^−1^ penicillin G sodium, and 0.025 mg mL^−1^ amphotericin B. Cells were maintained at 37 °C in a humidified atmosphere containing 5% CO_2_. For the assay, cells were detached by trypsinization and seeded in 24-well plates at a density of 3 × 10^4^ cells per well. After cell attachment, the hydrogel samples were placed in direct contact with the cells, and the final volume in each well was adjusted to 500 µL with complete culture medium. Untreated cells cultured in complete medium were used as the viability control. Triton X-100 at 2% (*v*/*v*) in phosphate-buffered saline was used as the cytotoxicity control, whereas pieces of polypropylene microcentrifuge tubes were included as a non-cytotoxic material control. After 24 h of exposure, the culture medium was removed and replaced with an MTT-containing solution. The cells were then incubated for 3 h at 37 °C under 5% CO_2_. Following incubation, 100 µL of the resulting solution from each well was transferred to a 96-well plate, and absorbance was measured at 530 nm using 590 nm as the reference wavelength in a Varioskan multimode microplate reader (Thermo Fisher Scientific, Waltham, MA, USA). Cell viability was calculated relative to the untreated control according to Equation (1):

(1) $$\mathrm{Cell}\;\mathrm{viability}\;(\%)=\frac{A_{\mathrm{sample}}-A_{\mathrm{blank}}}{A_{\mathrm{control}}-A_{\mathrm{blank}}}\times 100$$

where $A_{\mathrm{sample}}$ is the absorbance of cells exposed to the hydrogel, $A_{\mathrm{control}}$ is the absorbance of untreated cells, and $A_{\mathrm{blank}}$ is the absorbance of the cell-free assay medium.

### 2.7. Bioimaging Analysis

Bioimaging was conducted using confocal microscopy following a similar method described in the literature [4,47]. HeLa cells were plated on coverslips at 3 × 10^5^ cells/well density in a 6-well plate. After 48 h, the cells were treated with 400 μL of medium (DMEM + 10% SFB) and 1 cm^2^ of each hydrogel type (Table 1) for 30 and 60 min. Untreated cells with culture medium (DMEM + 10% SFB) were used as controls. Subsequently, the cells were fixed with 4% paraformaldehyde. The slides were mounted with Hydromount and analyzed using an Eclipse Ti confocal microscope (Nikon Instruments, Amstelveen, Netherlands) equipped with an oil-immersion objective (63× Plan-Apo/1.4 NA) and collected using a FITC filter with green emission (λexc = 447 nm and λem = 447–460 nm).

### 2.8. Antioxidant Activity of CMC–nGO Hydrogels

The antioxidant activity of CMC–nGO hydrogels was evaluated using the DPPH radical scavenging method [19]. The 2,2-diphenyl-1-picrylhydrazyl (DPPH) radical can accept electrons from antioxidant materials, forming a stable purple radical. This process results in scavenging of the radical, evidenced by a decrease in absorbance at 517 nm, allowing calculation of radical scavenging activity. The methodology involved dissolving 3 mg of DPPH in 100 mL of methanol, and hydrogels were added to 10 mL of the methanol solution and stirred magnetically for 4 h to ensure complete homogenization. The reaction was then allowed for 30 min at room temperature, protected from light, and the absorbance at 517 nm was measured. As a control for the effect of changes in DPPH, 100 µL of pure methanol was added to 900 µL of the DPPH–methanol solution. The free radical scavenging activity was calculated using Equation (2). Additionally, the analysis was tested in pure nGO as a comparison.

(2) $$DPPH\;scaveging\;activity=Inhibition\;\left(\%\right)=\frac{A_{0}-A_{1}}{A_{0}}\times 100\;$$

where $A_{0}\;$ is the absorbance of the control, and $A_{1}$ is the absorbance of each sample.

### 2.9. Antimicrobial Activity of CMC–nGO Hydrogels

The hydrogels based on nGO were tested against *E. coli* (ATCC 15597) and *C. albicans* (ATCC 90029). The inoculums of the test organism were incubated at 37 °C in Muller–Hinton medium until reaching the logarithmic phase, and the optical density of bacterial suspensions was measured at λ = 620 nm using a microplate reader (Spectra II Microplate Reader, Tecan, Mannedorf, Switzerland). The hydrogels were sterilized in an autoclave at 120 °C under 1 kgf.cm^−2^ for 15 min. Then, a disk of hydrogels with the same size (5 mm of diameter) as the antibiotic and antifungal disks was prepared. The antibiotics and antifungals used as positive controls (CP) were gentamicin (100 µg) and ketoconazole (100 µg), respectively. The negative control (S0) and pure nGO were also tested. After 24 h in the bacteriological oven at 37 °C, the halos (vertical and horizontal) were measured. The inhibition halo was measured in ImageJ software (version 1.54t) using the agar diffusion Petri dish image for each analysis to determine the diameter of the inhibition halo. All procedures were repeated in 5 microbial samples.

### 2.10. Statistical Analysis

Statistical analysis was conducted using one-way ANOVA with Tukey’s test (*p* < 0.05), using Origin v.8.1 software from OriginLab Corporation, Northampton, MA, USA.

## 3. Results and Discussion

The following sections discuss the physicochemical, electrochemical, and biological performance of the CMC–nGO formulations, with emphasis on composition–property relationships within the experimentally tested formulation window.

Within this context, CMC-based hydrogels containing nGO represent a promising biomaterial platform for cancer-related applications. By combining the structural and biocompatible properties of CMC with the intrinsic optical and biological functionalities of nGO, these nanocomposite hydrogels offer potential for fluorescence-assisted bioimaging, while simultaneously providing antioxidant and antimicrobial activities. Such multifunctionality is particularly relevant in tumour-associated microenvironments, where oxidative stress and microbial dysbiosis are known to contribute to disease progression [48]. Accordingly, CMC–nGO hydrogels were fabricated, comprehensively characterized, and evaluated regarding their physicochemical properties, cytocompatibility, fluorescence-assisted bioimaging capability, antioxidant performance, and antimicrobial activity.

### 3.1. nGO Characterization

**Figure 2:**
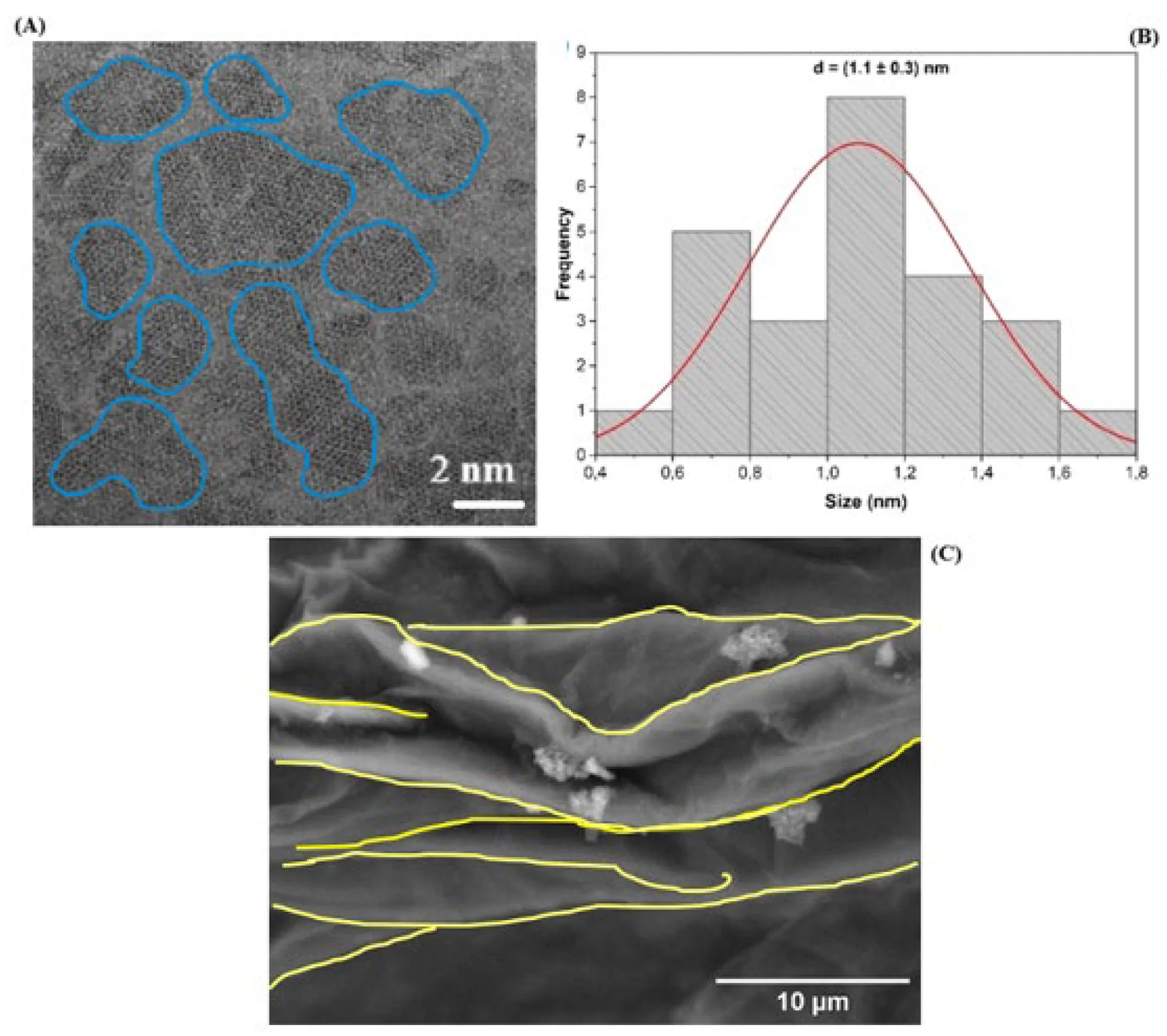
Physicochemical characterization of nanographene oxide (nGO). (**A**) TEM image showing nanosheet morphology; (**B**) size distribution of nGO nanosheets obtained by image analysis; (**C**) SEM image highlighting the characteristic wrinkled and folded surface structure. These features confirm the nanoscale morphology and high surface area of nGO.

Morphological characterization of nGO was carried out using different techniques. TEM analysis (Figure 2A) revealed a highly uniform nanosheet structure consistent with literature. ImageJ analysis of the TEM micrographs (Figure 2B) revealed an average nanosheet size of 2.0 ± 0.2 nm, in agreement with previously reported results [49].

The characterization of nGO was performed using Raman spectroscopy (Figure S1), and the spectrum exhibited three prominent bands: at 2698 cm^−1^, associated with the 2D-band, at 1594 cm^−1^, corresponding to the G band linked with graphite oxidation, attributed to the vibration of the sp^2^-C atom, and at 1349 cm^−1^ associated with the D band, related to an increase in sp^2^ size, probably because of extensive graphite oxidation [50,51]. Higher crystallization is associated with a higher ratio of this structure, although larger crystallites are related to a lower ratio of sp^2^ and sp^3^ structures. Moreover, D and 2D bands were observed, indicating a higher ratio of sp^2^ to sp^3^ structures and higher crystallization in this nGO [50,52,53,54], as the intense 2D band is associated with extensive oxidation [34]. Furthermore, the D-band is associated with aromatic rings that may introduce defects on the surface of nGO, possibly originating from OH ligands on the carbon (C) graphene surface [34]. In addition, the 2D band is attributed to double resonance transitions [54]. Although these features are consistent with the expected Raman characteristics of oxidized graphene derivatives, it is important to note that other factors, such as preparation-induced defects or environmental variations, may also influence peak intensity and position.

The SEM analysis (Figure 2C) revealed a surface morphology resembling crumpled paper, with characteristic wrinkles and folds. This feature is typically attributed to interactions among oxygen-containing functional groups—such as hydroxy (–OH), carbonyl (C=O), carboxylic acid (–COOH), and alcohol—commonly present in nGO [34,38,55].

Dynamic light scattering (DLS) analysis showed an average particle size of 5.0 ± 0.2 nm, which aligns with the supplier specifications. Additionally, EDX analysis (Figure S2) confirmed a predominant presence of carbon, while signals for oxygen, sodium, and aluminum were also detected likely due to some impurities in the graphite precursor used for nGO production.

Moreover, BET (Brunauer–Emmett–Teller) analysis specifically performed for nGO demonstrated an impressively large surface area, approximately 650 m^2^·g^−1^. This substantial surface area confers several advantages, such as enhanced adsorption capacity and faster diffusion rates, which are particularly beneficial in biological conditions [26,49], including biochemical reactions in physiological media or the controlled release of drugs [19,26,35].

### 3.2. CMC–nGO Hydrogel Characterization

The composition of hydrogels includes varying concentrations of CMC, citric acid, and nGO (Table 1). All samples contain 2% (*w*/*w*) CMC and 15% (*w*/*w*) citric acid, but the nGO content varies between samples. S0 contains no nGO, S1 contains 0.5% nGO, S2 contains 1% nGO, and S3 contains 3% nGO.

**Figure 3:**
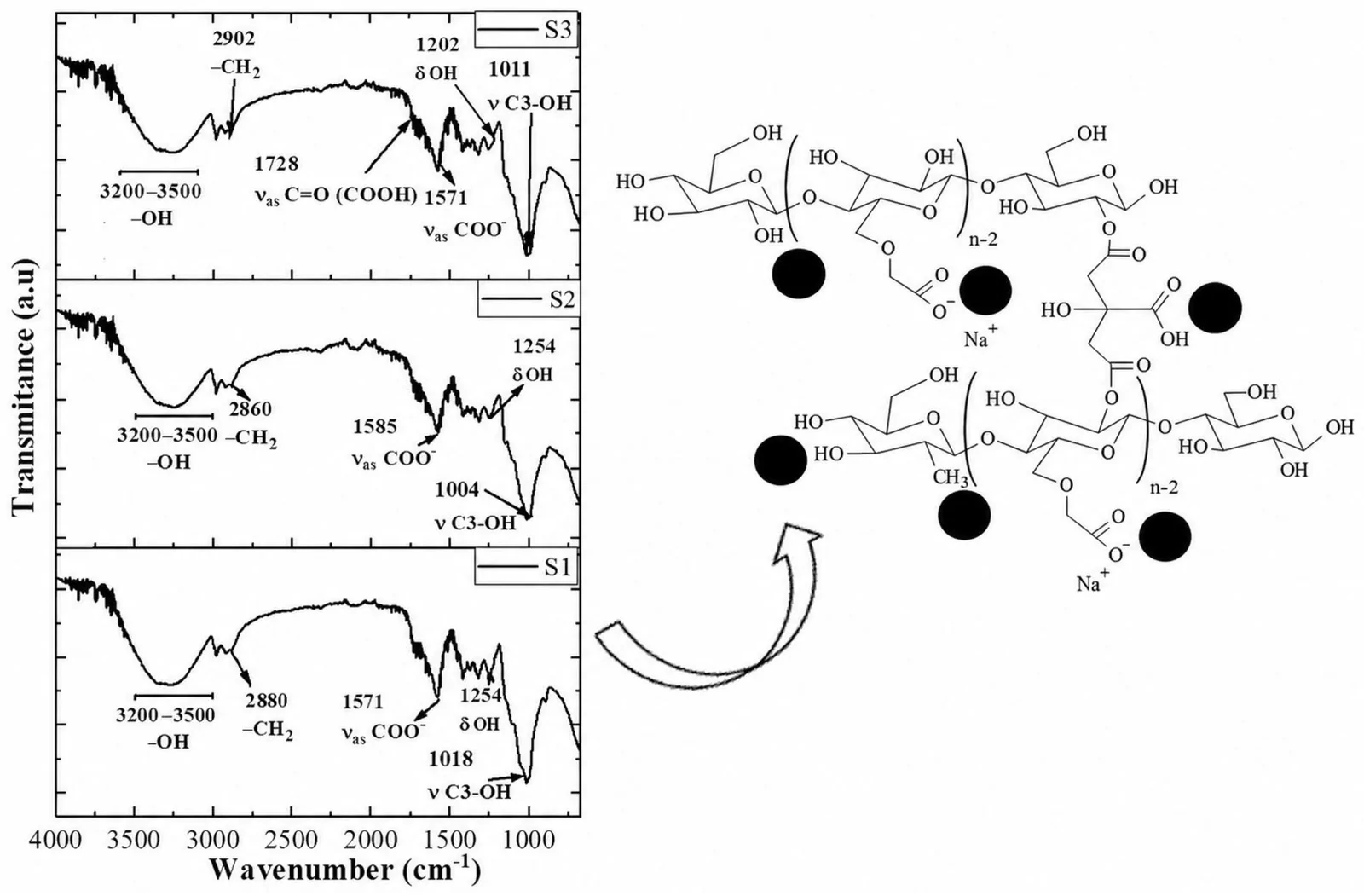
FTIR spectra of CMC–nGO hydrogels with increasing nGO content. Characteristic bands corresponding to hydroxyl (–OH), carbonyl (C=O), and carboxylate (COO^−^) groups are observed. Variations in band intensity and shifts indicate interactions between CMC and nGO, suggesting successful incorporation of nGO into the hydrogel network. The schematic representation illustrates the crosslinking reaction between CMC and citric acid in the presence of nGO.

FTIR analysis of the hydrogels (Figure 3) showed the expected functional groups of CMC and nGO. Bands in the 3200–3500 cm^−1^ region were assigned to –OH stretching and hydrogen-bonded hydroxyl groups [56,57,58]. For nGO (Figure S3), bands were observed at approximately 3270 cm^−1^ (O–H), 2974 cm^−1^ (CH_2_), 1733 cm^−1^ (C=O), 1621 cm^−1^ (C=C), and 1032 cm^−1^ (C–O) [56,57,58]. Changes in band intensity across the formulations are consistent with incorporation of oxygen-containing nGO into the CMC network. However, because the instrumental resolution was 4 cm^−1^, small peak displacements of only a few cm^−1^ should be interpreted cautiously and cannot, by themselves, demonstrate strong or specific chemical interactions.

Bands in the ~2860–2902 cm^−1^ region, assigned to CH_2_ stretching, showed small formulation-dependent displacements relative to the CMC hydrogel without nGO [59,60]. Given the 4 cm^−1^ FTIR resolution, these differences are treated as qualitative trends that may reflect changes in the local chemical environment rather than definitive evidence of a red shift or a specific interaction. Similarly, modest changes in the carboxylate region should be interpreted together with the overall spectral pattern and complementary structural characterization.

Bands associated with COO^−^ groups were observed in the ~1571–1590 cm^−1^ region, with changes in intensity as nGO content increased. These observations are compatible with involvement of carboxylate-containing environments in CMC–nGO association [43,61], but the small positional differences are close to the instrumental resolution and therefore do not establish a specific binding mechanism. Bands related to O–H and C–H bending of the polysaccharide ring near 1200 cm^−1^ and C–O vibrations from primary and secondary alcohols were also present in all samples [59,60,61,62].

SEM images (Figure S5) confirmed a dispersed distribution of nGO in the hydrogel matrix, with some clustering visible. These findings suggest that COOH and OH groups in the CMC chain play a crucial role in stabilizing and dispersing nGO within the hydrogel matrix, as supported by FTIR and Equation (3):

(3) $${CMC}_{COOH}^{:OH}\overset{pka\geq 4.5}{\to }{CMC}_{{COO}^{-}}^{:OH}+nGO\to [{CMC}_{{COO}^{-}}^{:OH}/nGO],$$

**Figure 4:**
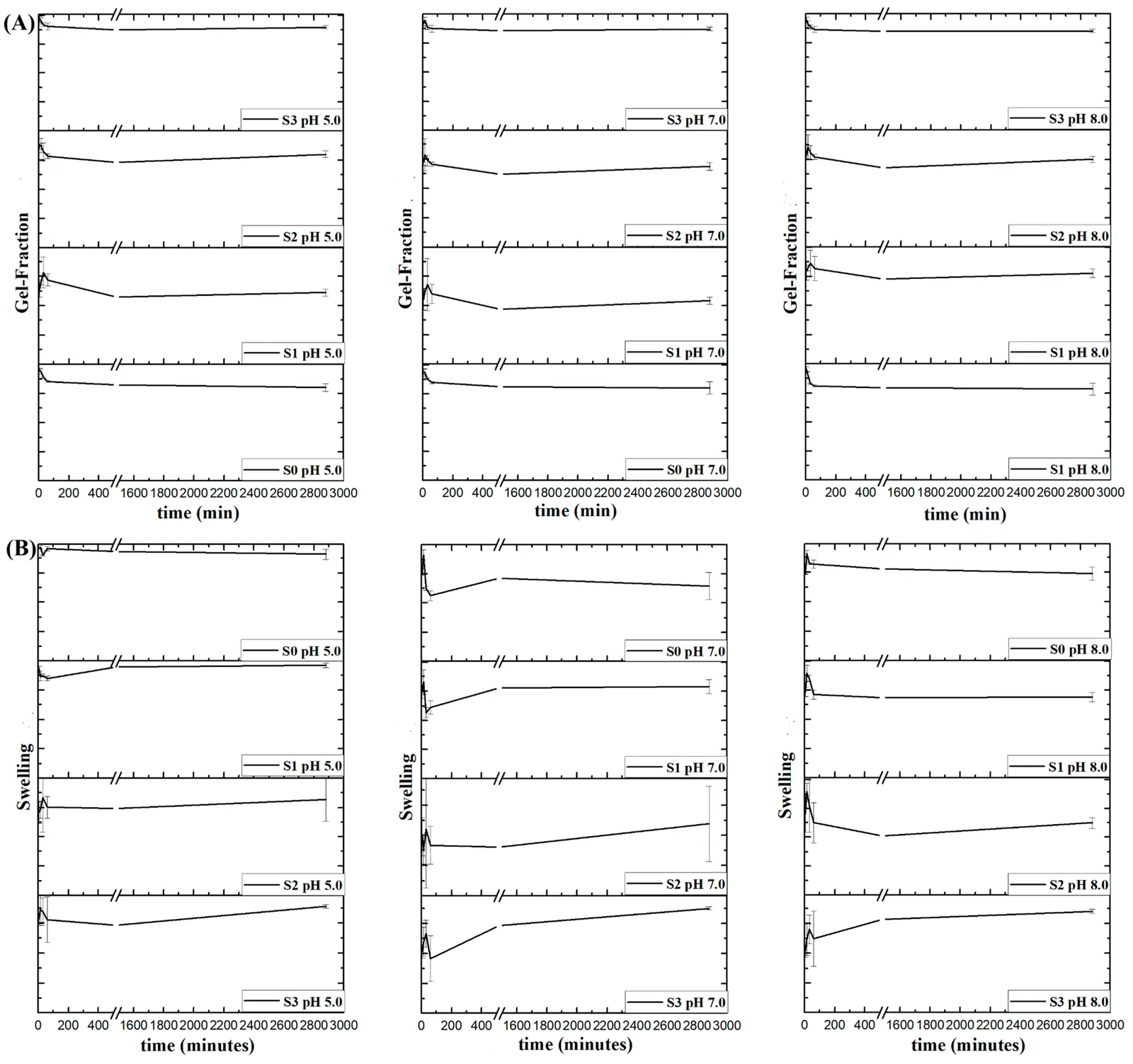
Swelling and gel fraction behavior of CMC–nGO hydrogels. (**A**) Gel fraction as a function of time and pH (5.0, 7.0, and 8.0); (**B**) swelling degree under the same conditions. The results demonstrate the influence of pH and nGO incorporation on hydrogel stability and water uptake capacity. Error bars represent standard deviation (*n* = 28).

The adsorption of fluids is a crucial property of biomaterials, and their dissolution/degradation behavior must be understood. Therefore, swelling and gel-fraction (dissolution) of the hydrogels were evaluated at pH 5.0, 7.0, and 8.0, conditions relevant to tumor microenvironments (Figure 4). For gel-fraction, all hydrogels exhibited statistically similar behavior (*p* > 0.05). A previous study reported that increasing GO led to a shift from low to high gel fraction [56]. Although this effect was not clearly observed here, the data suggest that increasing nGO may still influence crosslinking efficiency and hydrogel porosity, particularly under acidic conditions [4,35].

For swelling degree, nGO-containing hydrogels showed lower water uptake than S0 across the tested pH values, while differences among S1, S2, and S3 were not statistically significant (*p* > 0.05). At pH 5.0, S0 reached approximately 999 ± 5% swelling. This very high value should be interpreted cautiously: it reflects pronounced network expansion/water uptake under acidic conditions and may indicate reduced mechanical integrity of the CMC-only network rather than an intrinsically favorable property. The present swelling and gel-fraction measurements do not provide rheological confirmation of structural integrity at pH 5.0. Accordingly, future studies should correlate pH-dependent swelling with G′/G″ and directly assess possible nGO release or leaching under relevant conditions. Overall, pH strongly modulated swelling, whereas incorporation of nGO reduced the magnitude of water uptake within the tested formulation window [63].

**Figure 5:**
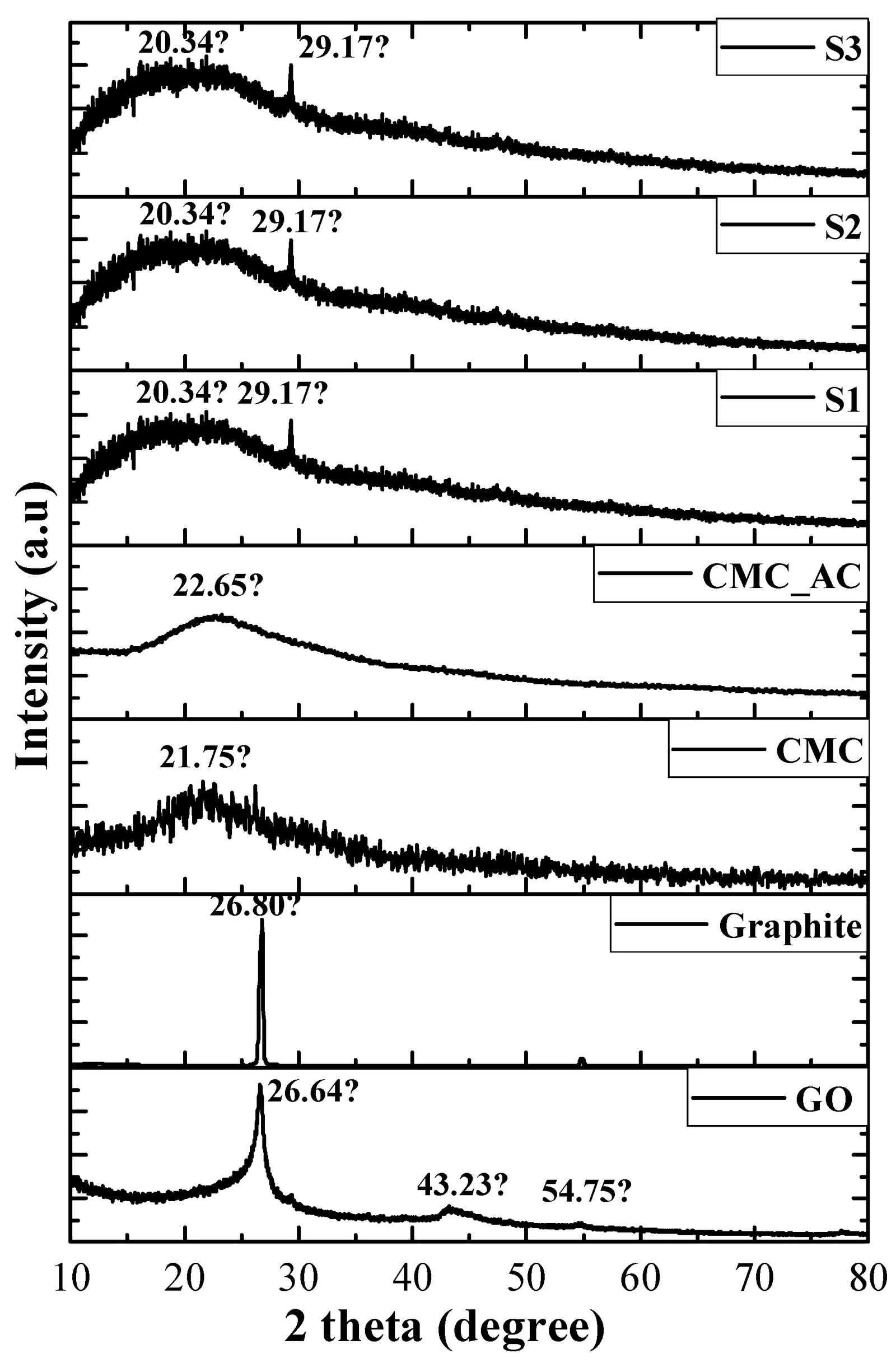
XRD patterns of graphite, graphene oxide (nGO), CMC, and CMC–nGO hydrogels. The incorporation of nGO and thermal crosslinking result in structural modifications and increased amorphization of the hydrogel matrix, confirming successful integration of nGO.

The XRD analysis (Figure 5) was performed on GO, graphite, CMC, and hydrogels. The incorporation of nGO into the hydrogel, together with thermal treatment, altered the crystalline structure. Cellulose I in both CMC and CMC/nGO showed peaks at 21.75° and 22.65°, respectively, with a slight shift in S0, likely due to citric acid crosslinking. Pure GO displayed a peak at 26.64° (incomplete oxidation of graphite) and another at 54.75° (reduction of oxygen groups) [34,38]. Graphite exhibited a strong peak at 26.80° (002 plane), also observed in GO, with a slight displacement. Hydrogels showed amorphization, likely due to low GO content (0.5, 1, 3%) and citric acid crosslinking. A single peak at 29.17°, shifted from GO and graphite, indicating nGO incorporation. No significant differences were observed among hydrogels. These results confirmed nGO incorporation and transition to a more amorphous structure [38].

**Figure 6:**
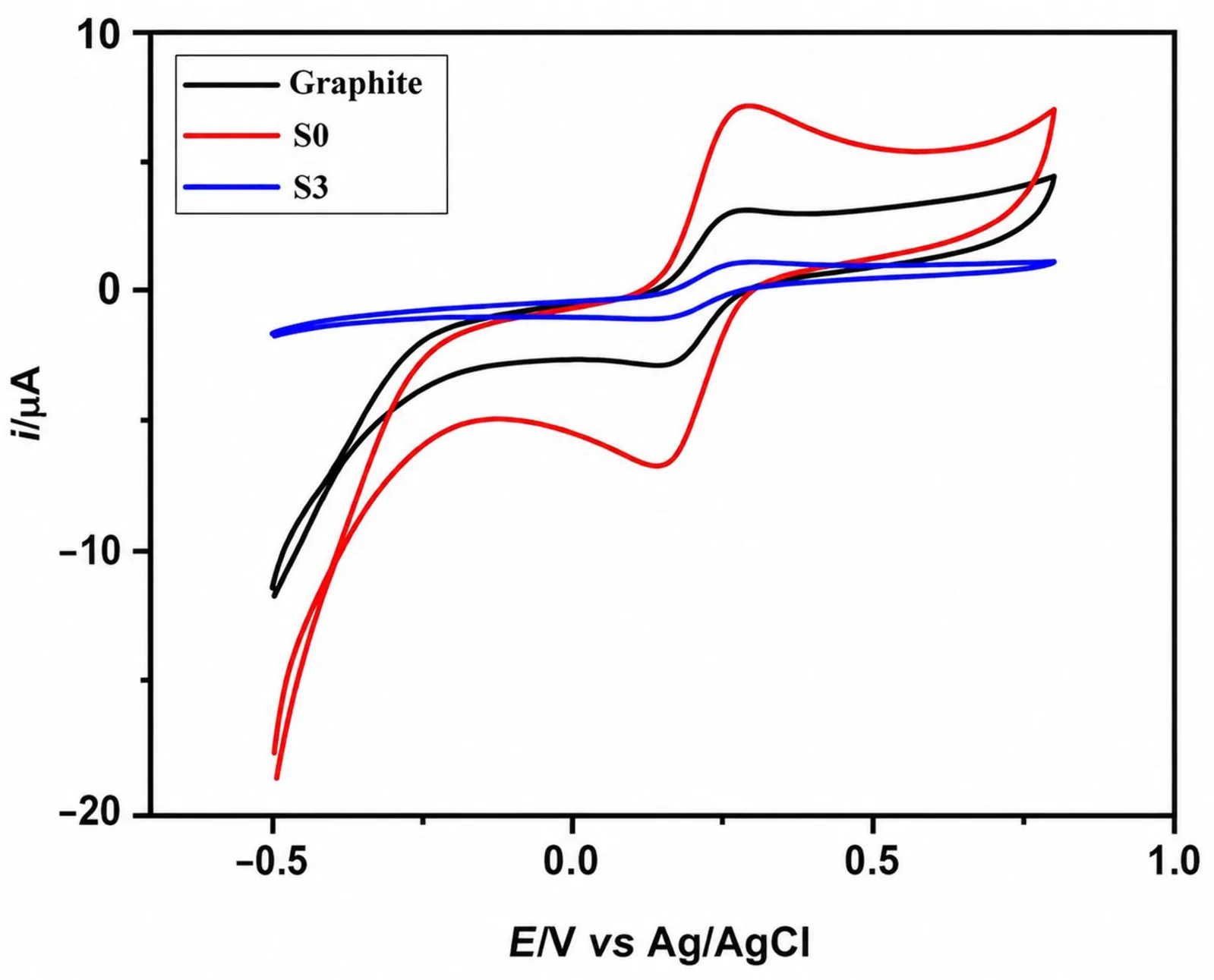
Electrochemical characterization of graphite electrodes. Voltammetric curves of bare graphite (black), graphite modified with CMC–citric acid hydrogel (blue), and graphite modified with nGO-loaded hydrogel (red). The enhanced current response indicates improved electrical conductivity and electron transfer associated with nGO incorporation.

To explore bioelectrical potential, the hydrogel S3, containing the highest nGO concentration, was used to modify a graphite electrode. The modified electrode produced a higher current response than bare graphite and the CMC–citric acid control (Figure 6), consistent with an altered electroactive interface and increased effective surface contribution [64,65,66]. Cyclic voltammograms were acquired for 10 consecutive cycles as described in Section 2.5. These repeated scans demonstrate short-sequence repeatability of the acquisition but should not be interpreted as a dedicated long-term stability test. Extended cycling, storage stability, current-retention analysis, and inter-electrode reproducibility will be required before claims regarding electrochemical durability can be made.

### 3.3. Biological Analysis

**Figure 7:**
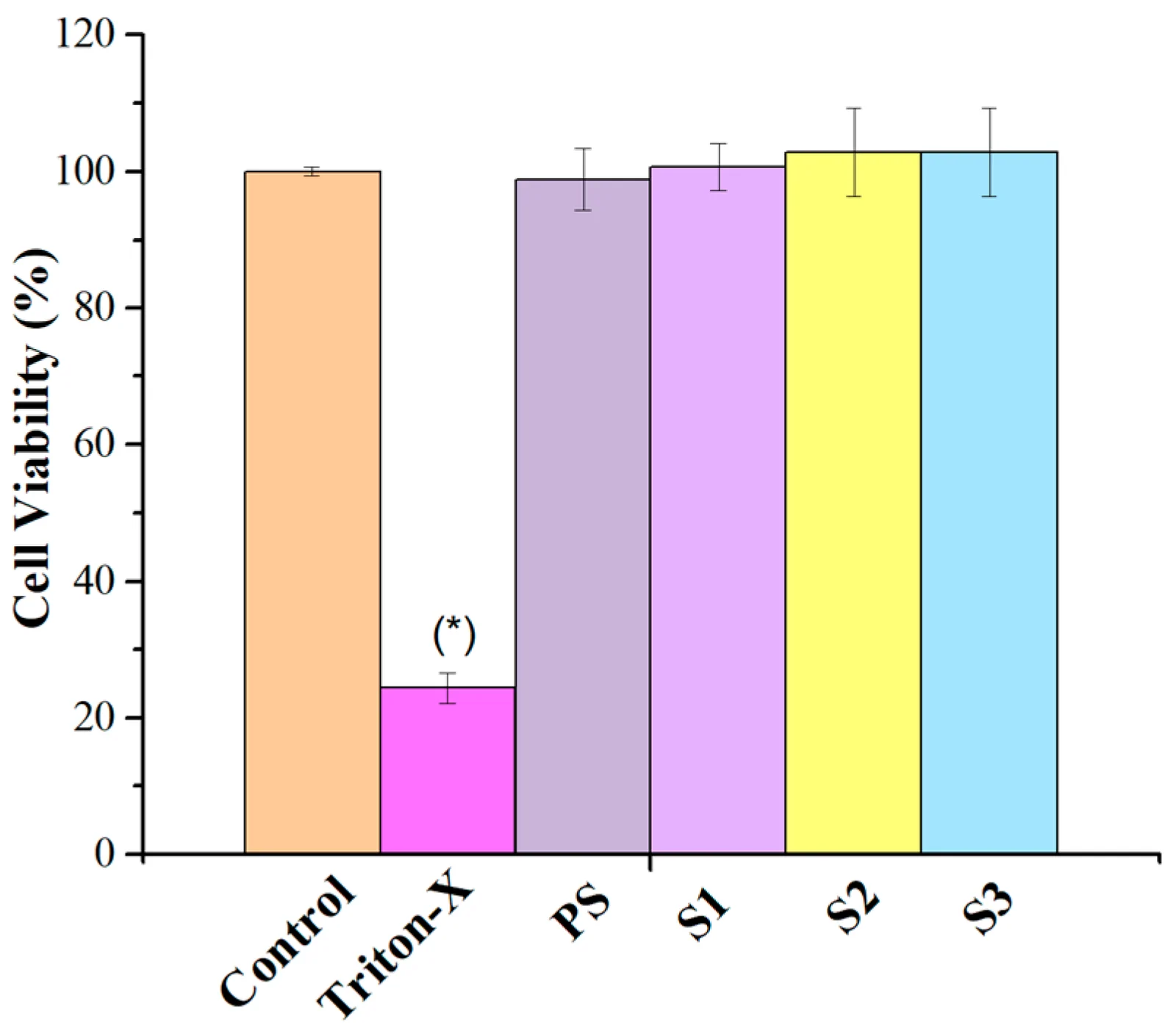
Cytocompatibility of CMC–nGO hydrogels evaluated in HeLa cells after 24 h of exposure using the MTT assay. Cell viability of hydrogel formulations (S0–S3) was compared with the negative control (PS), untreated control cells, and the positive control (Triton X-100). All hydrogel formulations maintained high cell viability (~90%), demonstrating excellent cytocompatibility. Data are presented as mean ± standard deviation. Statistical analysis was performed using one-way ANOVA (*p* < 0.05). The asterisk (*) indicates a statistically significant difference between the Triton X-100 group and all other groups.

The cellular viability upon exposure to CMC–nGO hydrogels was evaluated by MTT assay in HeLa cells (Figure 7). As expected, Triton X-100 produced a statistically significant reduction in viability relative to the untreated control (*p* < 0.05). In contrast, no statistically significant differences were observed between the hydrogel formulations and untreated cells (*p* > 0.05). Cell viability remained approximately 101 ± 3%, 103 ± 6%, and 103 ± 6% for S1, S2, and S3, respectively, supporting cytocompatibility under the tested 24 h in vitro conditions. These data do not address long-term exposure, biodegradation products, biodistribution, or immune responses, which require dedicated future studies.

Moreover, higher nGO content in hydrogels enhanced multifunctional behavior, attributed to more active binding sites and expanded surface area, promoting cell adherence [54]. However, it is important to emphasize that in vivo analysis is still required to confirm long-term biocompatibility, particularly regarding potential toxicity and immune responses. Previous studies have shown that nanomaterials can accumulate within biological systems over time, potentially leading to toxicity, and immune responses may vary depending on the host.

**Figure 8:**
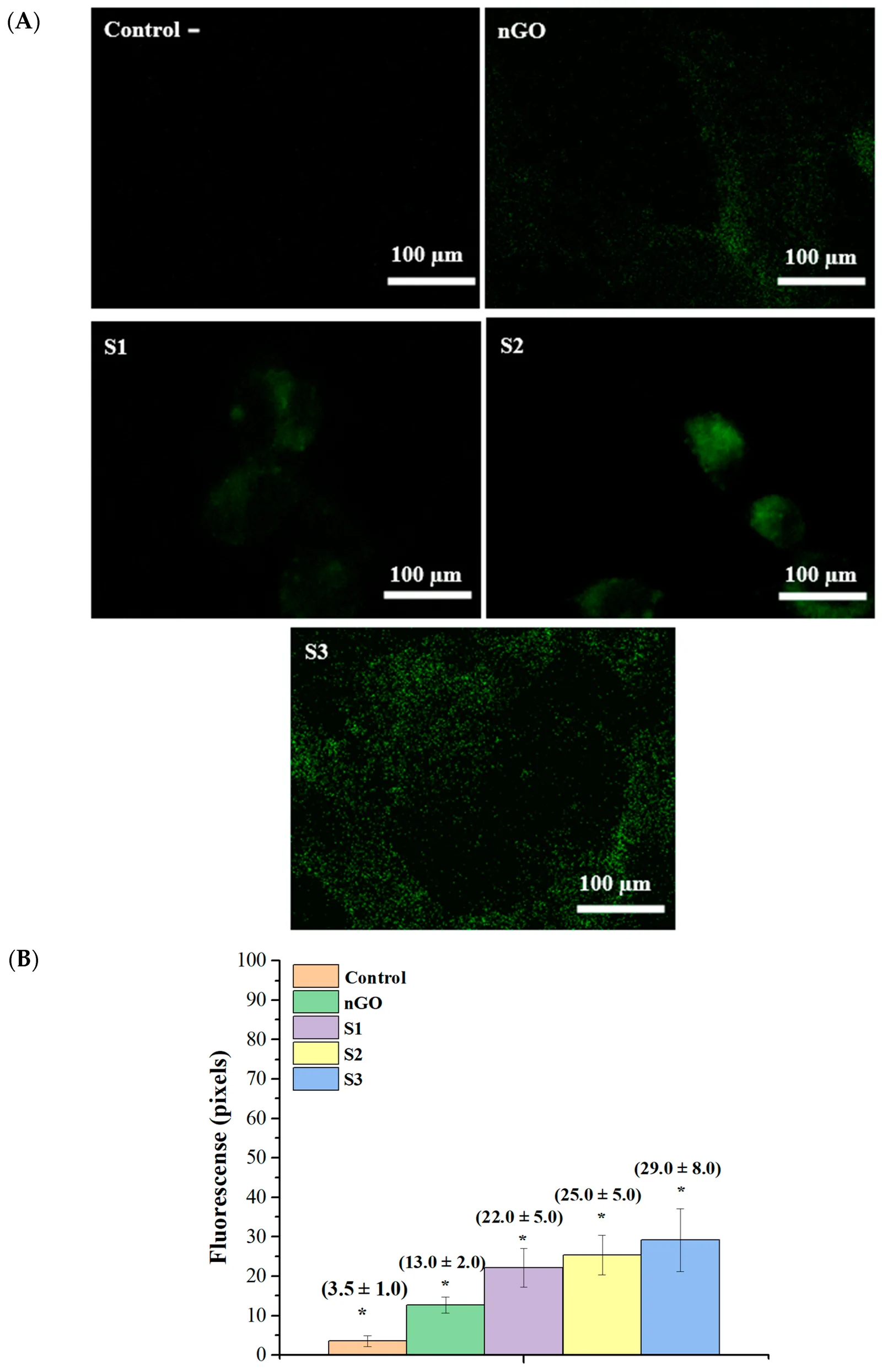
Fluorescence-assisted bioimaging of HeLa cells following exposure to CMC–nGO hydrogels. (**A**) Representative confocal microscopy images of untreated cells (control) and cells exposed to nGO and hydrogel formulations (S1–S3), demonstrating the intrinsic fluorescence of the nanocomposite hydrogels. (**B**) Quantitative analysis of fluorescence intensity, confirming enhanced fluorescence following exposure to nGO-containing hydrogels. These findings demonstrate the potential of CMC–nGO hydrogels as an intrinsically fluorescent platform for proof-of-concept HeLa cell bioimaging. Data are presented as mean ± standard deviation (*n* = 3). Statistical analysis was performed using one-way ANOVA (*p* < 0.05). The asterisk (*) indicates statistically significant differences between the indicated groups.

Confocal microscopy showed fluorescence-associated visualization of HeLa cells following exposure to nGO-containing formulations (Figure 8). Quantitative fluorescence-intensity analysis of the available confocal dataset (Figure 8B) confirmed an increased signal relative to untreated cells under the imaging conditions used. Nevertheless, this result should be interpreted as proof-of-concept fluorescence-assisted visualization rather than evidence of cancer-specific diagnostic capability. Only HeLa cells were evaluated, no non-cancerous cervical epithelial comparator was included, and the materials did not contain a cancer-specific targeting ligand. Consequently, the present data cannot distinguish selective cancer-cell recognition from non-specific cell/material association or uptake. Direct comparison with non-cancerous cervical epithelial cells (e.g., Ect1/E6E7), additional cancer cell lines, signal-to-background analysis, and targeting studies will be required to establish specificity.

The observed signal is consistent with a contribution from nGO and may also be influenced by the CMC/citric-acid network, since CMC has been described as a non-traditional luminogenic polymer [42]. However, photoluminescence spectra, quantum yield, fluorescence lifetime, excitation-dependent emission, and photostability were not measured in the present study. Therefore, the photophysical origin and relative contribution of nGO, the polymer network, and their interfacial interactions cannot be resolved from the current dataset. These measurements, together with quantitative time-resolved fluorescence analysis, are necessary in future work to establish the optical mechanism and performance of the platform.

Several other studies have demonstrated similar bioimaging strategies with carbon-based nanomaterials. Ahmed et al. (2023) [67] reported fluorescent carbon dots doped with silver ions that differentiated the luminescence intensity between normal (NIH3T3) and cancer cells (HeLa). Wanas et al. (2023) [68] developed a nanocomposite of graphene, folic acid, and ZnO for cancer bioimaging, showing green luminescence and selective targeting. Rodrigues et al. (2024) [4] produced a CMC–carbon dot hydrogel system for HeLa cell imaging, achieving a quatum yield of 17% and enhanced fluorescence. Together, these studies, along with the present findings, reinforce the potential of carbon-based nanomaterials, including carbon dots and graphene oxide, for bioimaging while also highlighting the need for comparative studies with normal cells to demonstrate diagnostic specificity.

### 3.4. Evaluation of the Antioxidant Activity of CMC–nGO Hydrogels

Oxidative stress is a biological imbalance linked to many diseases, including cancer, Alzheimer, Parkinson, cardiovascular, neurological, and respiratory disorders. It arises from excess free radical generation, which damages biomolecules and promotes pathological processes. The search for antioxidant compounds that can mitigate oxidative stress represents a promising approach to the prevention and therapy of cervical cancer [69].

**Figure 9:**
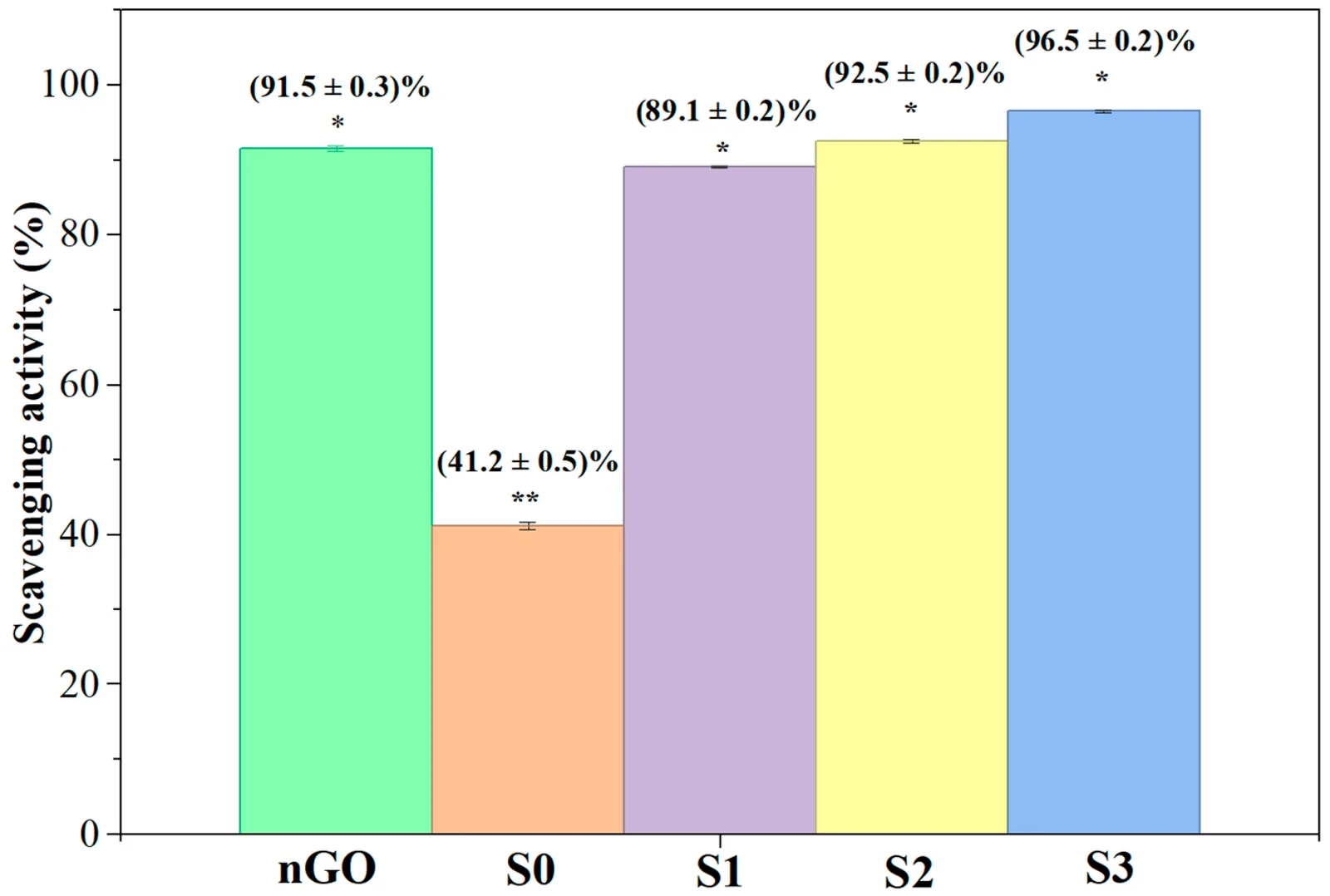
Antioxidant activity of CMC–nGO hydrogels determined by the DPPH radical scavenging assay. Hydrogels containing nGO exhibited significantly higher radical scavenging activity than the CMC-only hydrogel (S0), with antioxidant performance increasing as a function of nGO content. Data are presented as mean ± SD. Statistical analysis was performed using one-way ANOVA (*p* < 0.05). A single asterisk (*) indicates a statistically significant difference (*p* < 0.05), whereas a double asterisk (**) indicates a highly significant difference (*p* < 0.01).

Antioxidant agents can neutralize free radicals, thereby protecting cells [70]. In cancer, chromosomal instability and oncogene activation contribute to oxidative stress, which can be counteracted by antioxidant compounds. Substantial epidemiological evidence highlights their role in reducing disease incidence [71,72]. In this study, the antioxidant activity of nGO-based hydrogels was evaluated using the DPPH radical scavenging method (Figure 9) [73].

The DPPH assay determines the EC50, defined as the concentration at which 50% of radicals are scavenged. Higher EC50 indicates stronger antioxidant activity. All hydrogels with nGO (S1 (89.1 ± 0.2%), S2 (92.5 ± 0.2%), S3 (96.5 ± 0.2%)) and pure nGO ((91.5 ± 0.3%) displayed excellent antioxidant performance, comparable to natural products [72] and some essential oils [72,73]. The hydrogels containing nGO showed slightly stronger antioxidant activity than pure nGO, and both performed better than hydrogels without nGO (S0 (41.0 ± 0.5%)). This indicates that nGO is the main contributor to the antioxidant effect, while CMC provides structural support and may marginally enhance activity. Increasing nGO concentration within the hydrogel statistically enhanced antioxidant performance, demonstrating the key role of this nanomaterial. The graphene oxide is recognized as a versatile nanomaterial with broad biomedical, electrical, optical, and energy applications [34]. Although Figure 8A showed that pure nGO exhibited weaker luminescence than the nGO–CMC composite, its antioxidant properties remain dominant. The apparent discrepancy arises because luminescence and antioxidant activity are governed by different mechanisms: luminescence is stabilized by the CMC matrix, whereas radical scavenging is primarily driven by oxygenated functional groups on nGO. Collectively, these findings demonstrate the potential of CMC–nGO hydrogels as multifunctional biomaterials with intrinsic antioxidant activity and proof-of-concept fluorescence-assisted bioimaging capability.

### 3.5. Evaluation of the Antimicrobial Activity of Hydrogels Based on nGO

The microbiota in vaginal flora is highly important to promote protection against cervical cancer, HPV, and other types of cancer in the reproductive organs [9]. In addition, the presence of microorganisms, such as *Escherichia coli* and *Candida albicans*, promotes a competitive environment, leading to vaginal flora-induced changes in vaginal microbiota and dysbiosis [10,11]. In this context, the production of innovative multifunctional materials that protect against harmful microorganisms while demonstrating potential for cancer diagnosis and antioxidant activity is a highly promising alternative. Moreover, microorganisms provoke about 85% of mortality worldwide [49], and because fast-growing antimicrobial resistance is induced by indiscriminate abuse of antibiotics and antifungal drugs [26], eco-friendly and viable alternatives against these microorganisms are very interesting. In this sense, we propose an innovative CMC–nGO hydrogel as a multifunctional alternative against antimicrobial agents. Thus, based on the agar diffusion test, *E. coli* and *C. albicans* were tested as model microorganisms (bacteria and fungi) to assess antimicrobial activity.

**Figure 10:**
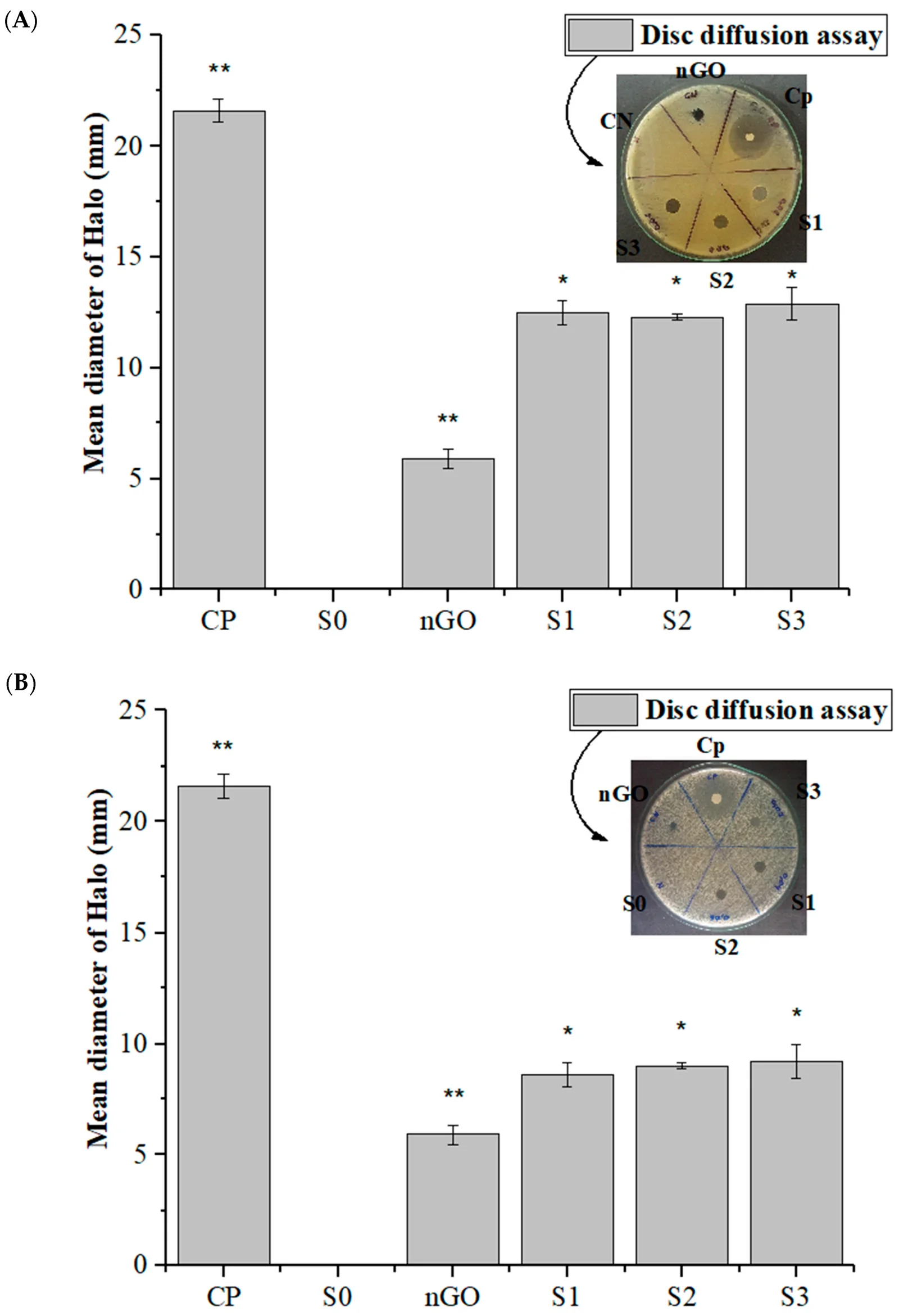
Antimicrobial activity of CMC–nGO hydrogels against (**A**) *Escherichia coli* and (**B**) *Candida albicans*, evaluated by the agar diffusion assay. The incorporation of nGO significantly enhanced the antimicrobial activity of the hydrogels, resulting in larger inhibition zones compared with the CMC-only hydrogel (S0). These findings further support the potential of CMC–nGO hydrogels as multifunctional bioactive biomaterials. Data are presented as mean ± SD. Statistical analysis was performed using one-way ANOVA followed by Tukey’s multiple comparisons test. A single asterisk (*) denotes *p* < 0.05, whereas a double asterisk (**) denotes *p* < 0.01.

The disk diffusion assay against *E. coli* revealed inhibition zones of 21.6 ± 0.5, 0, 5.9 ± 0.4, 12.5 ± 0.5, 12.3 ± 0.1, and 12.9 ± 0.8 mm for the positive control, S0, nGO, S1, S2, and S3, respectively (Figure 10A). The inhibitory action of hydrogels is evident, mostly caused by the incorporation of nGO, as the pure nGO exhibited an inhibitory zone. This action may be driven by the potential of hydrogels and nGOs to bind to bacterial cell surfaces via hydrogen bonds and oxygenated functional groups, thereby inducing physical damage to the bacterial cell wall [19,44]. For *C. albicans*, inhibition zones of 21.5 ± 1.0, 0, 4.91 ± 2.00, 8.6 ± 0.5, 9.0 ± 1.0, and 9.2 ± 0.9 mm were observed for the positive control, S0, nGO, S1, S2, and S3, respectively (Figure 10B). Similar to results against *E. coli*, an inhibitory effect of hydrogels is observed, likely due to physical damage to the fungal cell walls [19,44,74].

These results support the antimicrobial and antioxidant multifunctionality of the CMC–nGO system in vitro. However, they should not be interpreted as demonstrating cervical-cancer diagnosis or in vivo protection. The biological relevance of the antimicrobial findings will require validation in more representative microbial communities and biological models, while cancer-specific imaging will require appropriate non-cancerous comparators and targeting strategies.

A key limitation of the present work is that formulation development followed a one-factor-at-a-time strategy rather than a formal Quality by Design (QbD) framework. Thus, interactions among CMC concentration, nGO loading, mixing intensity and duration, citric-acid content, curing conditions, and environmental pH were not systematically mapped. The current results should therefore be interpreted as composition–property relationships within the tested processing window, not as an optimized design space. Future development should apply risk assessment and Design of Experiments (DoE) to relate these formulation/process variables to critical quality attributes including swelling and gel fraction, rheological and mechanical properties (LVR, G′, G″, yield stress and thixotropy), fluorescence performance and photostability, antioxidant and antimicrobial activity, cytocompatibility, and nanomaterial retention/release. Such studies should also directly evaluate whether prolonged high-speed mixing affects CMC molecular weight or viscosity.

## 4. Conclusions

This study establishes citric acid-crosslinked CMC–nGO hydrogels as sustainable, drug-free, and fluorophore-free biomaterial platforms that integrate optical, biological, and electrochemical functionalities within a single material. Incorporation of nGO reorganized the hydrogel network, reduced swelling, and modified its electrochemical response without compromising structural stability or cytocompatibility, with HeLa cell viability remaining at approximately 100% after 24 h. The nanocomposite hydrogels generated an intrinsic fluorescence signal approximately eightfold higher than that of untreated cells, enabling proof-of-concept fluorescence-assisted HeLa cell visualization without exogenous probes. They also exhibited radical-scavenging activity of up to 96.5% and antimicrobial activity against both *Escherichia coli* and *Candida albicans*, with maximum inhibition zones of 12.9 and 9.2 mm, respectively.

The principal novelty of this work lies in the synergistic combination of fluorescence-assisted cell imaging, antioxidant activity, antimicrobial performance, and electrochemical responsiveness in a simple CMC-based hydrogel containing no incorporated drug or conventional fluorophore. Rather than serving as a passive structural matrix, CMC supported and stabilized the functional contribution of nGO, resulting in a multifunctional biointerface with potential relevance to bioimaging, biosensing, and biomedical applications associated with cervical cancer. Nevertheless, the imaging findings should be interpreted as proof of concept rather than evidence of diagnostic specificity. Translation of this platform will require direct comparisons between cancerous and non-cancerous cervical cells, quantitative photoluminescence and photostability analyses, evaluation in three-dimensional and in vivo models, and investigation of long-term biocompatibility, biodegradation, biodistribution, and immune responses. Surface functionalization with disease-specific recognition ligands may further enable selective cellular targeting. Overall, these findings provide a robust foundation for the future development of multifunctional CMC–nGO biointerfaces that couple intrinsic imaging capability with biologically protective functions. In addition, the absence of rheological/mechanical characterization means that suitability for in vivo administration or long-term mechanical stability cannot be inferred from the present swelling and gel-fraction data alone.

## Figures and Tables

**Table 1:** Composition of the carboxymethyl cellulose–nanographene oxide (CMC–nGO) hydrogels. All formulations contained 2% (*w*/*v*) CMC and citric acid at 15% (*w*/*w*) relative to the CMC content, while the nGO concentration was varied from 0 to 3% (*w*/*w*) relative to CMC to investigate its influence on the physicochemical, electrochemical, and biological properties of the hydrogels.

Hydrogels	CMC (% *w*/*w*)	Citric Acid (% *w*/*w*)	nGO (%)
S0	2	15	0
S1	2	15	0.5
S2	2	15	1
S3	2	15	3

## Data Availability

The authors confirm that the data supporting the findings of this study are available upon request.

## Supplementary Materials

The following supporting information can be downloaded at: https://www.mdpi.com/article/10.3390/pharmaceutics18091121/s1, Figure S1. Raman spectrum of nanographene oxide (nGO), Figure S2. Energy-dispersive X-ray spectroscopy (EDX), Figure S3. FTIR spectrum of nanographene oxide (nGO) , Figure S4. FTIR spectrum of the CMC hydrogel crosslinked with citric acid (S0), Figure S5. Scanning electron microscopy (SEM) images of CMC-nGO hydrogels.
