# Supplementary material for: Multifunctional CMC–Nanographene Oxide Hydrogels Couple Fluorophore-Free Cancer Cell Imaging with Antioxidant and Antimicrobial Activity
Source: Pharmaceutics. 2026 Sep 7;18(9):1121. doi: 10.3390/pharmaceutics18091121 (PMC13610839; doi:10.3390/pharmaceutics18091121)
Supplement: Supplementary file 1 [file pharmaceutics-18-01121-s001.zip › pharmaceutics-4484524-supplementary.pdf]

Supplementary Materials

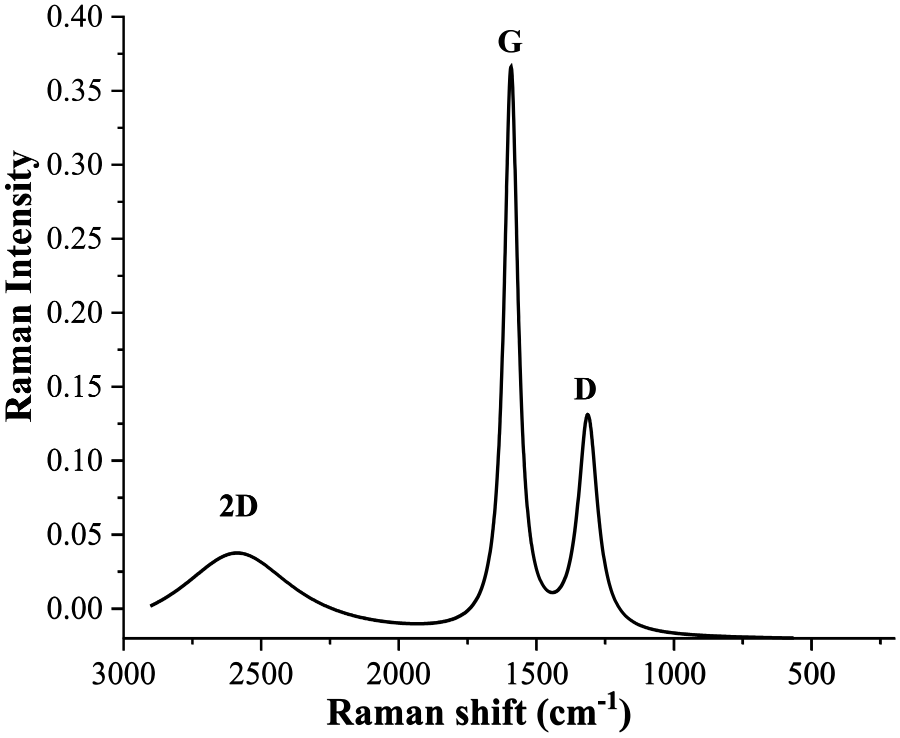

Figure S1. Raman spectrum of nanographene oxide (nGO) showing characteristic D, G, and 2D bands associated with structural defects, sp^2 carbon domains, and graphitic ordering. Peak deconvolution was performed using Lorentzian fitting, confirming the oxidized graphene structure and degree of disorder.

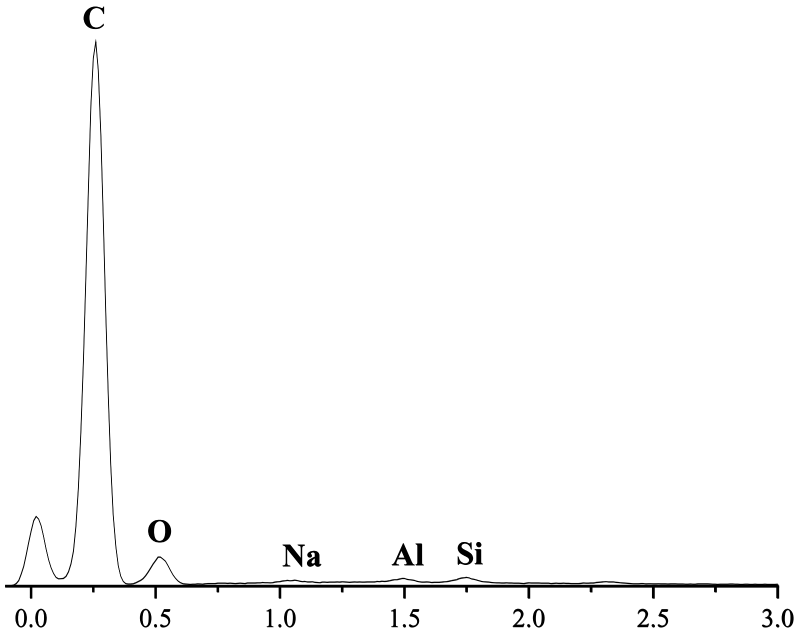

Figure S2. Energy-dispersive X-ray spectroscopy (EDX) analysis of nanographene oxide (nGO), confirming the predominant presence of carbon and oxygen elements, consistent with the chemical composition of oxidized graphene materials.

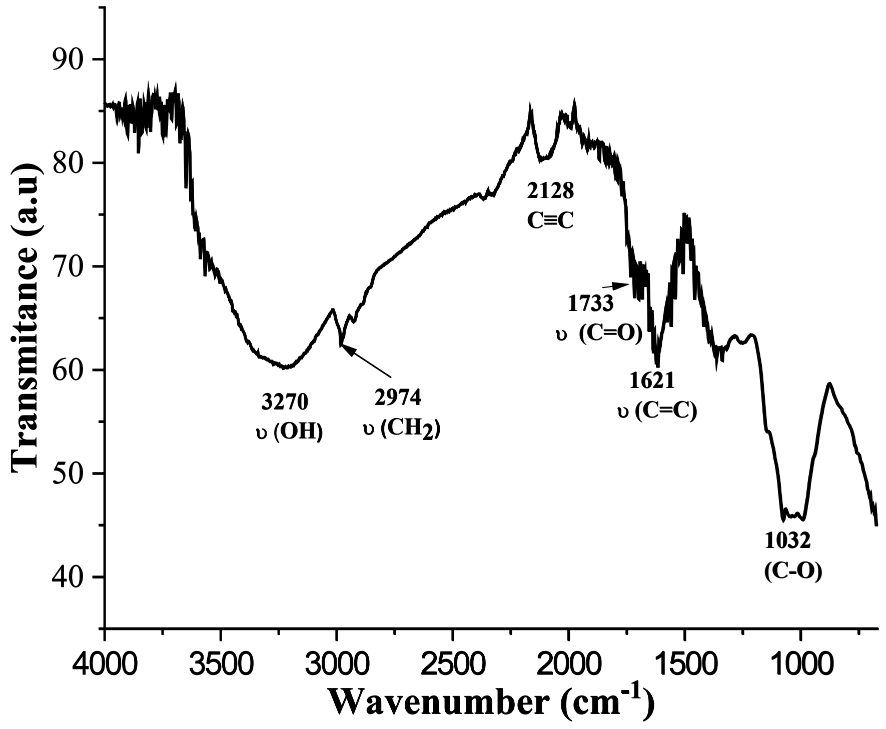

Figure S3. FTIR spectrum of nanographene oxide (nGO) highlighting characteristic functional groups, including hydroxyl (–OH) and carbonyl (C=O) vibrations, which contribute to its reactivity and interaction with the polymeric matrix.

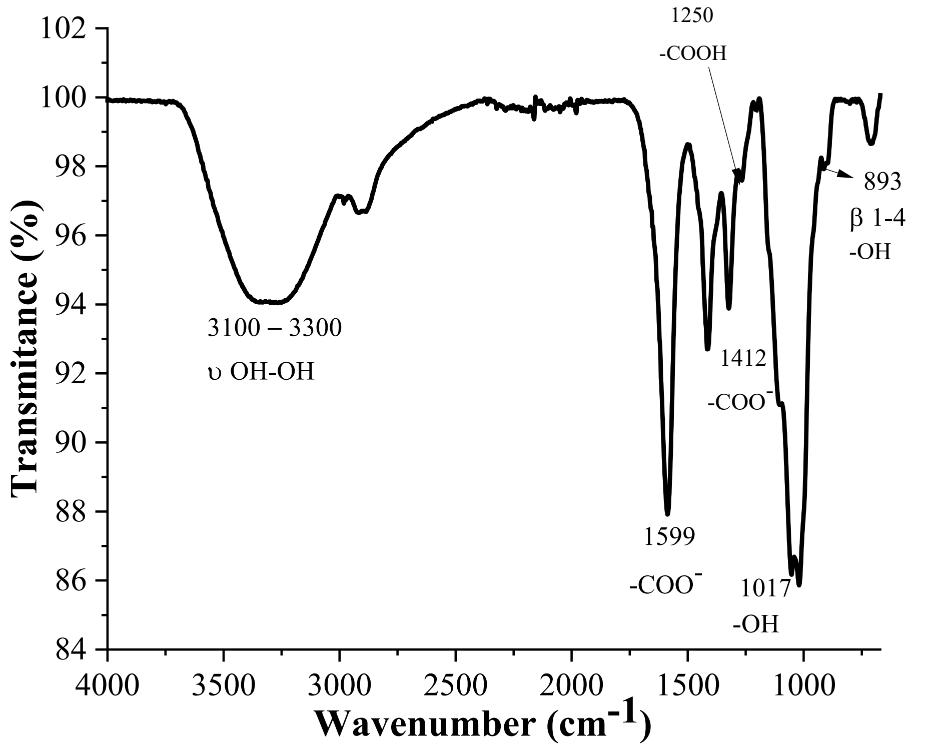

Figure S4. FTIR spectrum of the CMC hydrogel crosslinked with citric acid (S0), showing characteristic bands associated with polysaccharide structure and ester bond formation, confirming successful crosslinking in the absence of nGO.

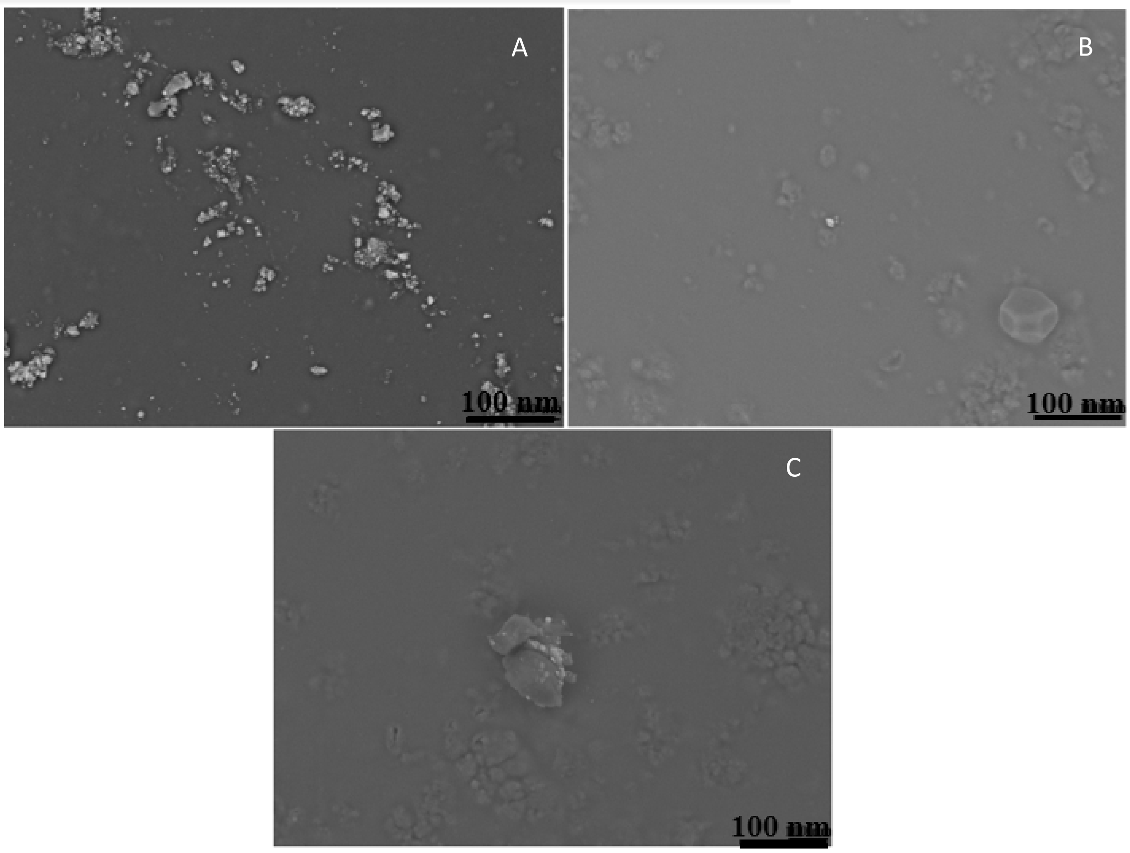

Figure S5. Scanning electron microscopy (SEM) images of CMC–nGO hydrogels: (A) S1, (B) S2, and (C) S3 (1000× magnification). The images reveal the surface morphology and distribution of nGO within the hydrogel matrix, highlighting changes in porosity and structural organization with increasing nGO content.
